# Malignant Disease of the Throat and Nose

**Published:** 1893-06

**Authors:** 


*Malignant Disease of the Throat and Nose.* By DAVID NEWMAN, M.D. Pp. x., 213. Edinburgh: YOUNG J. PENTLAND. 1892.

In the first part of this book Dr. Newman gives us a great deal of information on the diagnosis, prognosis, and treatment of œsophageal stricture. In the remaining part he treats of malignant disease of the larynx, tonsils, and nose in a way that shows he has had a good deal of practical experience of the cases about which he writes. All his statements are judiciously made, statistics are carefully compiled, numerous authors are quoted, and the clinical records of a good many patients are of much value. The book is very well worth reading.

